# Mental health burden and service barriers among medical and health sciences students: prevalence and determinants from a cross-sectional analysis

**DOI:** 10.3389/fpsyg.2026.1765447

**Published:** 2026-03-06

**Authors:** Shaimaa M. Hassan, Najeeb M. Alqahtani, Salihah M. Alshahrani, Aser M. Abuelnasr, Abdulkhalig A. Alhefzy, Khaled W. Al Assiri, Bandar W. Al Assiri, Omar S. Alharthi, Muhannad M. Alharbi

**Affiliations:** 1 Department of Histology, General Medicine Practice Program, Batterjee Medical College, Aseer, Saudi Arabia; 2 Histology and Cell Biology Department, Faculty of Medicine, Menoufia University, Shebin El Kom, Egypt; 3 General Medicine Practice Program, Batterjee Medical College, Aseer, Saudi Arabia; 4 Independent Researcher, Cairo, Egypt

**Keywords:** prevalence, determinants, depression, anxiety, stress, mental health services, medical and health sciences students

## Abstract

**Background:**

Medical and health sciences students face significant academic, clinical, and social pressures that increase their risk of psychological distress. Prior studies report elevated levels of depression, anxiety, and stress in this group. Barriers to accessing mental health services such as stigma, confidentiality concerns, limited awareness, and fear of negative consequences further compound this risk. Understanding the prevalence, determinants, and service barriers is essential for developing effective support systems.

**Aim:**

To determine the prevalence and determinants of depression, anxiety, and stress among medical and health sciences students at Batterjee Medical College, Saudi Arabia, and to identify barriers limiting their access to mental health services.

**Methods:**

A cross-sectional study was conducted among 384 students using a self-administered online survey. The Depression Anxiety Stress Scales-21 (DASS-21) assessed psychological distress, while the Barriers to Mental Health Services Scale-Revised (BMHSS-R) measured perceived obstacles to seeking help. Data were analyzed to identify prevalence, associated demographic and academic factors, and barriers to mental health service utilization.

**Results:**

Depression, anxiety, and stress (DAS) were evaluated using validated DASS-21 subscales demonstrating excellent internal consistency (*α* > 0.88). The total BMHSS score showed significant associations with academic factors, including major, year of study, and GPA (*p* ≤ 0.01). Gender was a strong predictor of psychological distress, with female students reporting significantly higher mean scores across all DAS domains (*p* ≤ 0.000439). Age was significantly associated with anxiety and stress (*p* ≤ 0.024). Students residing in urban areas reported higher anxiety scores than those in rural regions (*p* = 0.029). These findings identify gender, age, and residence as key demographic risk factors requiring targeted interventions.

**Conclusion:**

The study found a high prevalence of depression, anxiety, and stress among medical and health sciences students, with demographic and academic characteristics emerging as significant correlates. Intrinsic barriers, such as stigma and self-reliance, and extrinsic barriers, including accessibility and cost, further limited help-seeking and compounded psychological distress. These findings emphasize the need for comprehensive institutional mental health strategies and longitudinal research to enhance student wellbeing.

## Introduction

Mental health disorders represent a major global public-health concern and are increasingly recognized among university populations. Medical and health-sciences students face unique academic, clinical, and social stressors that place them at elevated risk for psychological distress. Depression, anxiety, and stress are among the most frequently reported conditions, with significant implications for academic performance, clinical competence, and future professional conduct (Peng et al., 2023; Li et al., 2022).

Recent meta-analytic data indicate that mental-health disorders are highly prevalent in this population. Jia et al. (2022) reported pooled global prevalence estimates of 48% for depression and 45% for anxiety among medical students during the COVID-19 pandemic (Peng et al., 2023). Similarly, Li et al. (2022) found that 39.4% of medical students exhibited depressive symptoms and 47.1% reported anxiety, both substantially higher than rates observed in non-medical peers. Stress, a critical antecedent and correlate of these conditions, remains pervasive; pooled prevalence across multiple studies exceeds one-third of respondents. These findings suggest that psychological distress among medical students is a persistent and systemic problem rather than a temporary pandemic-related phenomenon (Jabali, 2025).

Multiple factors contribute to the heightened vulnerability of medical and health-sciences students. Academic overload, competitive environments, exposure to illness and death, financial pressures, and limited leisure time have all been implicated (Mirza et al., 2021; Alwhaibi et al., 2023). Sociodemographic variables including gender, age, and living conditions also play significant roles. Female students and those in pre-clinical or early years of study consistently demonstrate higher levels of depression and anxiety (Cummerow et al., 2023; Kaiser et al., 2023). Moreover, inadequate sleep, poor coping mechanisms, and low perceived social support further exacerbate psychological strain (Qiu et al., 2024).

Despite the recognized burden of mental distress, utilization of professional mental-health services among medical students remains low worldwide (Al-Shahrani et al., 2023). The gap between perceived need and actual help-seeking is attributed to multiple intrinsic and extrinsic barriers. Intrinsic barriers include stigma, negative attitudes toward help-seeking, self-reliance beliefs, and lack of perceived need (Abdel-Hady et al., 2022; Mohebbi et al., 2023). Extrinsic barriers encompass limited-service availability, confidentiality concerns, time constraints, and financial or logistic challenges (Alsalman et al., 2024). A recent scoping review identified six recurring domains stigma, attitudinal resistance, confidentiality, time constraints, lack of awareness, and access difficulties that consistently inhibit help-seeking among medical trainees (Brar et al., 2024).

Within the Middle Eastern context, recent studies highlight similar challenges. In Saudi Arabia, Al-Zahrani et al. (2023) found that nearly three-quarters of healthcare students reported high stress levels, strongly associated with anxiety and depression (Alwhaibi et al., 2023). Likewise, Alsalman et al. (2024) reported that although 42.5% of medical students recognized a need for psychological care, only 16.2% accessed services, primarily due to stigma and attitudinal barriers. These findings underline the dual challenge faced by medical students: high psychological morbidity and under-utilization of available mental-health support.

Understanding the prevalence, determinants, and barriers to mental-health-service utilization among medical and health-sciences students is therefore critical for developing targeted interventions. Early detection and institutional support systems can mitigate the long-term consequences of psychological distress on both academic and clinical outcomes.

## Methodology

### Sampling method

A convenience sampling method was employed in this cross-sectional study. Students enrolled in the academic year 2025–2026 at Batterjee Medical College, across all levels, comprised the study population and were eligible to participate in this study.

### Study area

This research study was conducted at Batterjee Medical College in Saudi Arabia, with a focus on undergraduate students.

### Study subjects

The study population comprised students enrolled at Batterjee Medical College during the academic year 2025–2026, representing all levels of study.

Inclusion criteria:

1. Undergraduate students who join any BMC program in the 2025 to 2026 academic year.
2. Students agree to take part and give their informed consent.
3. Students can complete the survey.

Exclusion criteria:

Students with a documented history of psychiatric diagnoses (such as depression, anxiety disorders, bipolar disorder, schizophrenia). Additionally, participants are currently receiving psychiatric treatment or counseling, including pharmacological or psychological therapy.

### Sample size

The sample size was calculated using the Raosoft sample size calculator^1^ as the minimally required sample size. With a total population of 7,217 students, a 95% confidence interval level, a 5% margin of error, and an estimated prevalence of 60.8% for stress, anxiety, and depression among medical students in Saudi Arabia, the calculated sample size was 349. To account for an anticipated 10% nonresponse rate, the 384 participants were adjusted as the final sample size.

### Study design

This research study was a cross-sectional study conducted among students enrolled at Batterjee Medical College during the academic year 2025–2026, across all levels.

### Data collection tool

The data used in this study were collected from students using a structured, self-administered online questionnaire structured to estimate symptoms of depression, anxiety, and stress, along with perceived barriers to accessing mental health care among medical students. There are two versions of the questionnaire: the Arabic and English versions of the validated Depression, Anxiety, and Stress Scale (DASS-21). This scale has demonstrated strong internal consistency, with Cronbach’s alpha coefficients of 0.88 for depression, 0.88 for anxiety, and 0.89 for stress in prior validation studies. Moreover, the Barriers to Mental Health Services Scale-Revised (BMHSS-R) was used to identify perceived obstacles to seeking professional psychological support.

The questionnaire has three sections. The first one includes the collected sociodemographic data, such as age, gender, academic specialization, year of study, current GPA, residence, and marital status.

The second section employed the DASS-2 (Gomez, 2016). The DASS-21 is a self-report instrument comprising 21 items, divided evenly into three subscales assessing depression, anxiety, and stress symptoms over the past week. Each item is rated on a 4-point scale ranging from 0 (“did not apply to me at all”) to 3 (“applied to me very much or most of the time”). For each subscale, scores are summed and then multiplied by two to enable interpretation equivalent to the original DASS-42 scale.

Interpretation followed established severity cutoffs for each domain:

Depression: [0–9] normal, [10–13] mild, [14–20] moderate, [21–27] severe, [≥28] extremely severe.

Anxiety: [0–7] normal, [8–9] mild, [10–14] moderate, [15–19] severe, [≥20] extremely severe.

Stress: [0–14] normal, [15–18] mild, [19–25] moderate, [26–33] severe, [≥34] extremely severe.

The third section utilized the Barriers to Mental Health Services Scale Revised (BMHSS-R). BMHSS-R is a multidimensional self-report instrument designed to assess perceived obstacles to mental health service utilization, particularly among older adults. The scale comprises 10 subscales, each representing a barrier supported by mental health services literature. The BMHSS-R is organized into two categories: intrinsic barriers (including seeking help attitudes, stigma, knowledge, and fear of psychotherapy sessions, inability to find a psychotherapist, thoughts that depressive symptoms are normal) and extrinsic barriers (including insurance and payment concerns, ageism, concerns about psychotherapist qualifications, physician counselling, and the transportation availability). The response format is modified from a 5-point to a 4-point Likert scale (Pepin et al., 2015).

### Data collection process

The study used an online self-reported questionnaire to collect data. The research team shared the survey link on popular social networks, such as Twitter and WhatsApp, which the target group frequently used. Before participating, potential respondents received a detailed document that explained the research aims, study objectives, and the requirements of their participation.

## Statistical techniques

All statistical analyses will be performed using the Statistical Package for the Social Sciences (SPSS) Version 25.0 (IBM Corp., Armonk, NY, USA). Descriptive statistics will be calculated for sociodemographic factors and all questionnaire items. Continuous data will be represented as means and standard deviations, while categorical variables will be presented as frequencies and percentages. The Kolmogorov–Smirnov test will be used to determine the normality of the continuous variables. The Kruskal-Wallis and Mann–Whitney *U* tests will be used to evaluate the association between the independent variables and the continuous outcome variables, or Pearson’s chi-square (χ2) test if the outcome is categorical.

For normal distributions, an independent sample *t*-test will be employed to determine the significance of mean differences. In situations where the distribution is not normal, the Mann–Whitney *U* test will be used. Similarly, to compare the means of three or more categories, either an analysis of variance technique (for normally distributed data) or the Kruskal-Wallis test (for non-normally distributed variables) will be applied.

### Ethical approval

All participants provided written informed consent prior to data collection, having been fully informed about the study’s aims, methods, and potential risks. Ethical approval was obtained from the Research Ethics Committee at Batterjee Medical College (Reference No. RES-2025-0066), ensuring that the study adhered to rigorous ethical protocols.

## Results

**Figure 1:**
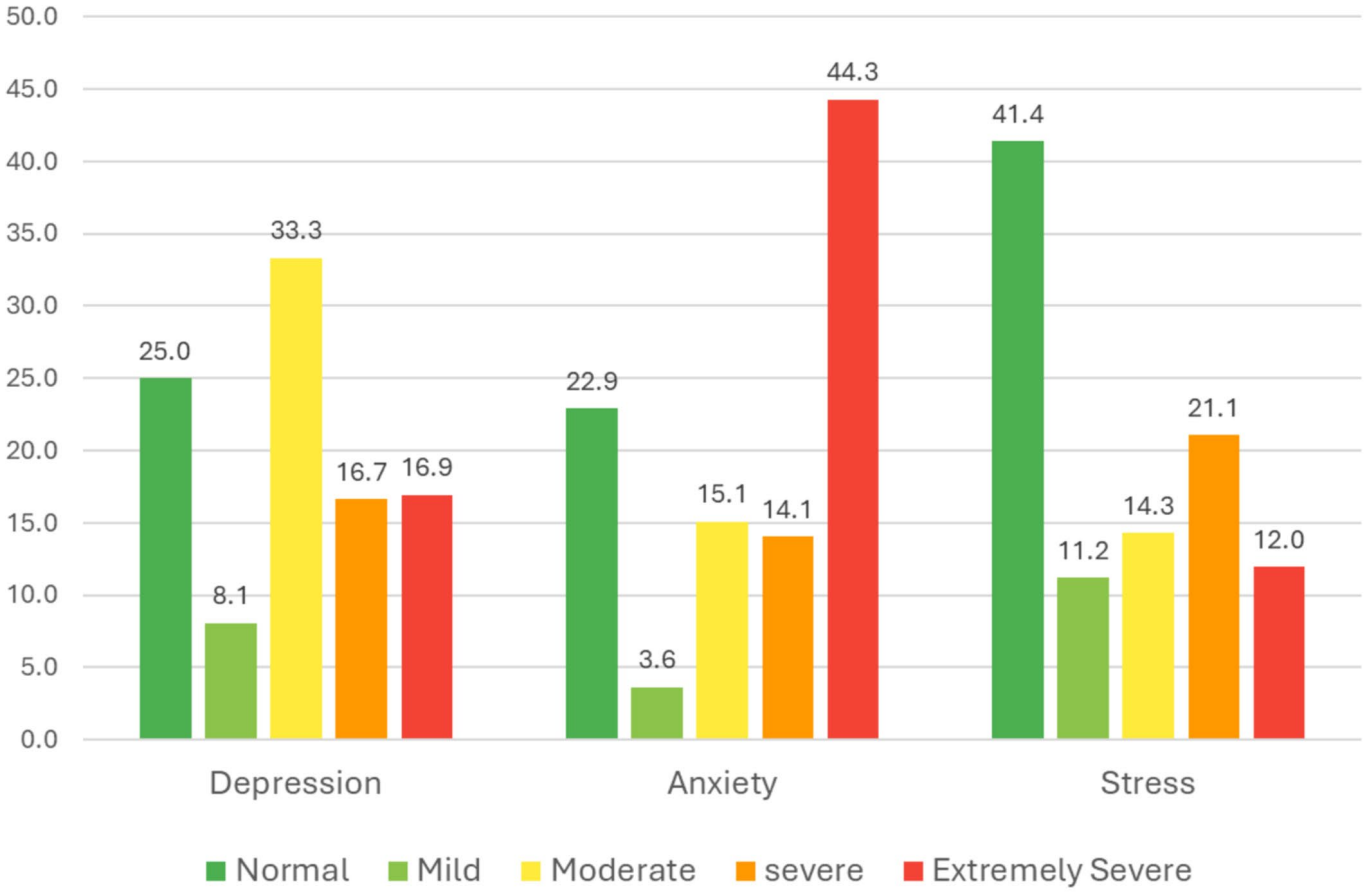
Prevalence of depression, anxiety and stress among the study participants.

The current study showed a substantial prevalence of depression, anxiety, and stress symptoms among the participants. A depression scale showed that 25% of the individuals fell within the normal range, while the majority of participants, 33.3% are in the moderate range. The proportion is almost the same for the severe and extremely severe categories at 16.7 and 16.9%, respectively. Particularly, the lowest range is the mild stage, 8.1%. Similarly, for stress, 41.4% of individuals are normally categorized, while the severe category ranks second in proportion, with 21.1%. The mild, moderate, and extremely severe categories are almost identical, with percentages of 11.2, 14.3, and 12%, respectively. The anxiety scale is different from the other two scales, depression and anxiety. Almost half of the participants, 44.3%, are categorized as extremely severe, while 3.6% are considered to be in the mild range. The participants are categorized as normal, with 22.9%, while the moderate and severe categories are 15.1 and 14.1%, respectively (Figure 1).

The depression scores for females (mean = 18.71) show a statistically significant difference from those for males (mean = 14.58) among participants (*p* = 0.0004). There is no statistical difference in depression scores among age groups (*p* = 0.062), the majors of participants (*p* = 0.194), the study year (*p* = 0.124), and the GPA of students (*p* = 0.068). Regarding the place of residence and marital status, there is no significant difference (*p* = 0.436) and (*p* = 0.75), respectively (Table 1).

**Table 1:** Relation between depression with sociodemographic characteristics (*n* = 384).

	Depression				*p-*value^U/K^
	Mean	SD	Median	IQR	
Gender					
Male	14.58	9.913	14	16	0.000439^**^
Female	18.71	10.873	18	14	
Age					
18–22	17.25	10.271	16	14	0.062^*^
23–27	15.89	11.184	16	18	
28–30	19.55	11.807	19	11	
31–35	19.58	8.319	19	12	
36–40	12.26	10.274	14	20	
Major					
Medicine	16.29	10.332	14	18	0.194
Dentistry	14.3	8.853	14	12	
Pharmacy	23.56	10.76	22	20	
Nursing	17.86	9.789	18	13	
Occupational therapy	13.33	11.719	18	-	
Respiratory therapy	18.27	11.516	21	19	
Physical therapy	16.29	15.568	10	27	
Healthcare administration	20.19	8.316	18	8	
Other	14.78	12.173	14	18	
Study year					
1st year	16.62	11.127	18	18	0.124
2nd year	18.78	10.753	16	12	
3rd year	17.15	10.973	18	17	
4th year	17.08	9.969	18	10	
5th year	13.33	10.042	13	18	
GPA					
Below average	18.96	10.324	20	12	0.068
Good	19.37	9.257	18	9	
Very good	18.88	10.071	18	11	
Excellent	16.10	10.487	16	18	
Outstanding	15.31	11.073	14	18	
Residence					
Village	15.5	11.155	17	20	0.436
City	17.02	10.525	16	14	
Marital status					
Single	16.88	10.644	16	15	0.750
Married	16.75	10.847	18	17	
Divorced	14	9.043	15	17	

^U^Mann–Whitney U test, ^K^Kruskal–Wallis test. IQR = interquartile range, SD = standard deviation. * Statistical significance *p* > 0.05, ** very highly statistically significant *p* > 0.001.

The gender category shows a statistically significant difference (*p* < 0.001) in anxiety scores between females (mean = 20.8) compared to males (mean = 14.28). The age groups reveal a significant difference in anxiety scores (*p* = 0.024). Regarding the majors of participants (*p* = 0.353), study year groups (*p* = 0.259), GPA of students (*p* = 0.224), and marital status (*p* = 0.310), there are no statistically significant differences in anxiety scores. The place of residence shows statistically significant differences in anxiety scores (*p* = 0.029) (Table 2).

**Table 2:** Relation between anxiety with sociodemographic characteristics (*n* = 384).

	Anxiety				*p-*value^U/K^
	Mean	SD	Median	IQR	
Gender					
Male	14.28	9.943	14	14	0.000^**^
Female	20.8	11.473	22	17	
Age					
18–22	19.01	11.235	18	18	0.024^*^
23–27	16.55	11.292	18	20	
28–30	19.73	12.256	22	19	
31–35	18.25	9.962	17	15	
36–40	11.57	9.438	14	18	
Major					
Medicine	18.49	11.607	18	20	0.353
Dentistry	14.37	9.688	14	14	
Pharmacy	21.33	10.1	20	14	
Nursing	17.84	9.924	19	13	
Occupational therapy	14	12.49	18	–	
Respiratory therapy	19.27	11.679	24	22	
Physical therapy	15.43	15.22	11	27	
Healthcare administration	19.81	9.272	18	7	
Other	15.41	12.151	16	21	
Study year					
1st year	17.18	10.227	18	17	0.259
2nd year	19.8	11.796	18	18	
3rd year	18.15	11.352	18	19	
4th year	17.86	11.22	18	17	
5th year	14.97	11.202	14	20	
GPA					
Below average	18.67	11.476	20	24	0.224
Good	19.07	8.199	18	9	
Very good	20.24	11.069	18	15	
Excellent	17.38	11.025	18	18	
Outstanding	16.49	11.952	18	20	
Residence					
Village	14.93	10.346	16	18	0.029^*^
City	18.28	11.353	18	20	
Marital status					
Single	18.19	11.331	18	18	0.310
Married	15.94	11.081	14	18	
Divorced	16.20	9.496	20	10	

^**U**^Mann–Whitney U test, ^**K**^Kruskal–Wallis test. IQR = interquartile range, SD = standard deviation. * Statistical significance *p* > 0.05, ** very highly statistically significant *p* > 0.001.

Similar to depression and anxiety scores, there is a statistically significant difference in stress scores between the female (mean = 22.47) and male (mean = 14.92) in the gender group (*p* > 0.001). Between the age groups, there is a statistically significant difference (*p* = 0.007). The age group 31–35 reveals a higher stress score (mean = 23.67) compared with the age groups 23–27 (mean = 17.02) and 36–40 (mean = 13.57), respectively. While the age groups 18–22 and 28.30 show the same stress score (mean = 20.08) and (mean = 20.45), respectively. The majors of participants, study years of students, GPA of students, place of residence, and marital status do not show statistical differences in stress scores among participants (Table 3).

**Table 3:** Relation between stress with sociodemographic characteristics (*n* = 384).

	Stress				*p-*value^U/K^
	Mean	SD	Median	IQR	
Gender					
Male	14.92	9.78	14	16	0.000^**^
Female	22.47	11.828	22	18	
Age					
18–22	20.08	11.701	18	20	0.007^*^
23–27	17.02	11.478	16	16	
28–30	20.45	11.126	20	13	
31–35	23.67	9.504	22	15	
36–40	13.57	9.908	14	20	
Major					
Medicine	20.04	12.356	18	22	0.157
Dentistry	14.52	8.64	14	12	
Pharmacy	20.89	11.05	16	18	
Nursing	18.14	9.805	18	14	
Occupational therapy	12	10.583	16	-	
Respiratory therapy	21.4	12.07	24	18	
Physical therapy	17.29	14.647	13	24	
Healthcare administration	21.24	9.284	20	12	
Other	17.07	11.748	16	16	
Study year					
1st year	18.3	11.307	18	20	0.143
2nd year	20.73	11.409	18	16	
3rd year	19	11.65	20	19	
4th year	19.84	11.029	16	15	
5th year	15.67	12.286	15	22	
GPA					
Below average	20.22	11.318	22	20	0.421
Good	18.24	10.48	14	17	
Very good	20.88	11.525	20	14	
Excellent	19.52	10.735	20	16	
Outstanding	17.86	12.199	17	21	
Residence					
Village	16.33	11.727	17	20	0.061
City	19.43	11.459	18	16	
Marital status					
Single	19.37	11.712	18	17	0.285
Married	17.66	10.8	16	17	
Divorced	14	9.8	16	21	

^**U**^Mann–Whitney *U* test, ^**K**^Kruskal–Wallis test. IQR = interquartile range, SD = standard deviation. * Statistical significance *p* > 0.05, ** very highly statistically significant *p* > 0.001.

The severity of depression scores started from normal, with 55.2% of normal scores being outstanding students, 26% being excellent students, and the below-average, good, and very good students are 4.2, 4.2, and 10.4%, respectively. The mild score of depression is equally prevalent among excellent and outstanding students, at 32.3%. Meanwhile, the scores below average, good, and very good are 9.7, 6.5, and 19.4%, respectively. The moderate score of depression is high in outstanding 39.1%, and the lowest in below-average 5.5%. The scores of good, very good, and excellent groups are 18.8, 20.3, and 16.4%, respectively. The severe score of depression is highest in the outstanding group, at 42.2%, and is 21.9% in the excellent group, while the good group shows the lowest score, at 4.7%. The scores of below-average and very good groups are 12.5 and 18.8%, respectively. The scores of extremely severe depression are highest in the outstanding group 40% and lowest in the below-average group, 7.7%. The scores of good, very good, and excellent groups are 12.3, 18.5, and 21.5%, respectively. There is a statistically significant difference across GPA groups (*p* = 0.012) (Table 4).

**Table 4:** Association of depression severity with GPA (*n* = 384).

GPA	Depression												*p*-value
	Normal		Mild		Moderate		Severe		Extremely severe		Total		
	*N*	*%*	*N*	*%*	*N*	*%*	*N*	*%*	*N*	*%*	*N*	*%*	
Below average	4	4.2	3	9.7	7	5.5	8	12.5	5	7.7	27	7	0.012^*^
Good	4	4.2	2	6.5	24	18.8	3	4.7	8	12.3	41	10.7	
Very good	10	10.4	6	19.4	26	20.3	12	18.8	12	18.5	66	17.2	
Excellent	25	26	10	32.3	21	16.4	14	21.9	14	21.5	84	21.9	
Outstanding	53	55.2	10	32.3	50	39.1	27	42.2	26	40	166	43.2	

* Statistically significance *p* > 0.05.

The severity of anxiety scores ranged from normal, with 58% of normal scores being outstanding, followed by 22.7% being excellent. The percentages of students categorized as below average, good, and very good are 6.8, 2.3, and 10.2%, respectively. Among those with mild anxiety, 35.7% were rated excellent and 28.6% outstanding. The lowest percentage, 7.1%, falls within the below-average group category, while both the good and very good groups have a percentage of 14.3%. The moderate score of anxiety is high in outstanding 41.4%, and the lowest in below-average 5.2%. The scores of the good and very good groups are the same, at 15.5%, while the excellent group scores 22.4%. The severe score of anxiety is highest in the outstanding group, at 33.3%, and is 27.8% in the excellent group. The below-average group shows the lowest score, at 3.7%, while the good and very good scores are 22.2 and 27.8%, respectively. The scores of extremely severe anxiety are highest in the outstanding group, 40.6% and lowest in the below-average group, 8.8%. The scores of good, very good, and excellent groups are 9.4, 18.2, and 22.9%, respectively. There is a statistically significant difference across GPA groups (*p* = 0.009) (Table 5).

**Table 5:** Association of anxiety severity with GPA (*n* = 384).

GPA	Anxiety												*p*-value
	Normal		Mild		Moderate		Severe		Extremely severe		Total		
	*N*	*%*	*N*	*%*	*N*	*%*	*N*	*%*	*N*	*%*	*N*	*%*	
Below average	6	6.8	1	7.1	3	5.2	2	3.7	15	8.8	27	7	0.009^*^
Good	2	2.3	2	14.3	9	15.5	12	22.2	16	9.4	41	10.7	
Very good	9	10.2	2	14.3	9	15.5	15	27.8	31	18.2	66	17.2	
Excellent	20	22.7	5	35.7	13	22.4	7	13	39	22.9	84	21.9	
Outstanding	51	58	4	28.6	24	41.4	18	33.3	69	40.6	166	43.2	

* Statistical significance *p* > 0.05.

The severity of stress scores ranged from normal, with 47.8% of normal scores being outstanding, followed by 19.5% being excellent. The percentages of students categorized as good and very good are equally proportionate, at 13.2%, while the below-average group has the lowest score, at 6.3%. Among those with mild stress, 37.2% were rated outstanding, while the lowest percentage 7%, falls within the below-average group category. The scores of the below-average, good, and very good groups are 9.3, 25.6, and 20.9%, respectively. The moderate score of stress is high in the outstanding category at 36.4%, and the lowest in the below-average category at 5.5%. The scores of the good, very good, and excellent groups are 9.1, 21.8, and 27.3%, respectively. The severe stress score is highest in the outstanding group, at 42%, and is 27.3% in the excellent group. The good group shows the lowest score, at 8.6%, while the below-average and very good scores are equally proportionate 12.3%. The scores for extremely severe stress are highest in the outstanding group, at 43.5%, and lowest in the below-average group, at 2.2%. The scores of good, very good, and excellent groups are 8.7, 26.1, and 19.6%, respectively. There is no statistically significant difference across GPA groups (*p* = 0.371) (Table 6).

**Table 6:** Association of stress severity with GPA (*n* = 384).

GPA	Stress												*p*-value
	Normal		Mild		Moderate		Severe		Extremely severe		Total		
	*N*	%	*N*	%	*N*	%	*N*	%	*N*	%	*N*	%	
Below average	10	6.3	3	7	3	5.5	10	12.3	1	2.2	27	7	0.371
Good	21	13.2	4	9.3	5	9.1	7	8.6	4	8.7	41	10.7	
Very good	21	13.2	11	25.6	12	21.8	10	12.3	12	26.1	66	17.2	
Excellent	31	19.5	9	20.9	15	27.3	20	24.7	9	19.6	84	21.9	
Outstanding	76	47.8	16	37.2	20	36.4	34	42	20	43.5	166	43.2	

Descriptive analysis and reliability (Cronbach’s alpha) were calculated for the Depression Anxiety Stress Scales – 21, the BMHSS-R total scores and subscale scores (Tables 7, 8). Jugessur (2022), states that alpha values of 0.70 or higher show strong reliability. Each subscale, Depression, Anxiety, and Stress, has an alpha of 0.9, indicating high reliability (Jugessur, 2022).

**Table 7:** The reliability of depression anxiety stress scales – 21 (*n* = 384).

Scales	Number of items	Cronbach’s alpha	*M* (SD)
Depression	7	0.885	16.79 (10.63)
Anxiety	7	0.881	11.26 (17.76)
Stress	7	0.893	18.95 (11.54)

**Table 8:** The reliability of barriers to mental health seeking services scale revised (*n* = 384).

Subscale/scale items	Alpha	*M* (SD)
Intrinsic barriers (internal factors)		
Help-seeking attitudes 1,7,10	0.72	1.99 (0.77)
Stigma 2, 3, 8	0.67	2.01(0.76)
Knowledge and fear of psychotherapy 6,11,	0.69	2.17 (0.85)
Belief about inability to find a psychotherapist 13,21, 23	0.85	2.28 (0.85)
Belief that symptoms are “normal” 4, 5,	0.61	2.27 (0.81)
Extrinsic barriers		
Insurance/payment concerns 12, 24	0.82	2.68 (1)
Ageism 14, 22, 27, 28, 29, 30	0.89	2.03 (0.75)
Concerns about therapist qualifications 9, 16, 17, 18, 19, 20, 26, 31–44	0.93	2 (0.73)
Transportation concerns 15, 25	1	2.41 (1)
Total intrinsic barriers (internal factors)	0.843	2.14 (0.63)
Total extrinsic barriers (internal factors)	0.827	2.28 (0.72)
Total extrinsic barriers	0.97	2.28(0.72)

Six subscale scores had a Cronbach’s alpha above 0.70, while the stigma, knowledge, fear of psychotherapy, and belief that symptoms are normal subscales did not meet this cut-off (Table 9). The Intrinsic, Extrinsic, and overall Total Scores showed excellent internal consistency. Each subscale showed a statistically significant and positive correlation with the others and with the total score. The insurance, stigma, and beliefs that symptoms are normal subscales were only weakly to moderately related. Some relationships were very strong, with correlation values above 0.69. There was also a strong correlation between total intrinsic and total extrinsic barriers.

**Table 9:** The correlations for the total score of barriers to mental health seeking services scale revised and subscale (*n* = 384).

Subscale/scale items	Help-seeking	Stigma	Knowledge & Fear of psychotherapy	Finding a therapist	Belief that symptoms are normal	Insurance/payment	Ageism	Confidence in therapist’s qualifications	Transportation	Intrinsic	Extrinsic	BMHSS-R total Sc
Help-seeking	1.00											
Stigma	0.734	1.00										
Knowledge and fear of psychotherapy	0.622	0.634	1.00									
Inability to find a therapist	0.450	0.487	0.558	1.00								
Belief that symptoms are “normal”	0.466	0.440	0.421	0.370	1.00							
Insurance/payment concerns	0.257	0.288	0.421	0.575	0.425	1.00						
Ageism	0.556	0.578	0.678	0.639	0.360	0.426	1.00					
Concerns about therapist qualifications	0.572	0.629	0.735	0.624	0.391	0.417	0.877	1.00				
Transportation concerns	0.326	0.358	0.384	0.730	0.321	0.560	0.535	0.502	1.00			
Total Intrinsic Barriers (Internal Factors)	0.832	0.836	0.826	0.723	0.669	0.481	0.700	0.743	0.510	1.00		
Total Extrinsic Barriers	0.501	0.545	0.642	0.786	0.440	0.749	0.827	0.813	0.824	0.724	1.00	
BMHSS-R Total Score	0.683	0.717	0.775	0.818	0.570	0.669	0.830	0.842	0.729	0.905	0.940	1.00

*r* = 0.30 medium, *r* = 0.50 large.

Table 10 shows that, according to the BMHSS-R score, there is no statistically significant difference among gender groups, age groups, or marital status groups. The BMHSS-R score shows statistically significant differences among the majors of participants (*p* = 0.006), the study year groups (*p* = 0.01), the GPA groups (*p* = 0.002), and the place of residence groups (*p* = 0.028).

**Table 10:** Relation between BMHSS-R with sociodemographic characteristics (*n* = 384).

	BMHSS-R total score		*p-*value
	*N*	%	
Gender			
Male	179	46.6	0.899
Female	205	53.4	
Age			
18–22	191	49.7	0.225
23–27	124	32.3	
28–30	22	5.7	
31–35	24	6.3	
36–40	23	6	
Major			
Medicine	164	42.7	0.006^*^
Dentistry	27	7	
Pharmacy	9	2.3	
Nursing	74	19.3	
Occupational Therapy	3	0.8	
Respiratory Therapy	30	7.3	
Physical Therapy	14	3.6	
Healthcare Administration	21	5.5	
Preparatory	1	0.3	
Other	41	10.7	
Study year			
1st year	61	15.9	0.01^*^
2nd year	79	20.6	
3rd year	82	21.4	
4th year	102	26.6	
5th year	60	15.6	
GPA			
Below average	27	7	0.002^*^
Good	41	10.7	
Very good	66	17.2	
Excellent	84	21.9	
Outstanding	166	43.2	
Residence			
Village	60	15.6	0.028^*^
City	324	84.4	
Marital status			
Single	310	80.7	0.244
Married	64	16.7	
Divorced	10	2.6	

* Statistical significance *p* > 0.05, ** very highly statistically significant *p* > 0.001.

## Discussion

This cross-sectional study investigated the prevalence and determinants of depression, anxiety, and stress (DAS) and the associated barriers to accessing mental health services among medical and health sciences students at Batterjee Medical College in Saudi Arabia. The findings reveal a significant mental health burden within this student population and identify key demographic and academic factors influencing psychological distress and help-seeking behaviors. The following discussion interprets these results within the context of the existing literature.

### High prevalence of psychological distress

The current study demonstrates a profoundly concerning picture of psychological distress among medical and health sciences students at Batterjee Medical College. The data indicates a substantial and severe prevalence of depression, anxiety, and stress (DAS) symptoms, with anxiety prevalence demonstrating disproportionately elevated levels relative to other domains, indicating a critical area of concern. Nearly half of the participants (44.3%) reported anxiety levels in the “extremely severe” range according to the DASS-21 criteria. This is an exceptionally high prevalence that surpasses many previous reports and signals a critical situation requiring immediate institutional attention. To place this in context, a meta-analytic study by Puthran et al. (2021) reported a global pooled prevalence of depression among medical students at 27.2%, a figure already considered high, yet the severity distribution in our sample where only 25% fell within the normal range for depression suggests an even more acute localized crisis (Puthran et al., 2016).

The pattern of distress observed is multifaceted. For depression, the distribution was predominantly moderate (33.3%), severe (16.7%), and extremely severe (16.9%) categories, collectively indicating that two-thirds of the student population was experiencing clinically significant depressive symptoms. The stress profile was comparably concerning, with 41.4% of participants classified within the normal range, while 21.1% demonstrated severe symptomatology. This triad of high prevalence across all three domains is a hallmark of the medical student experience, but its intensity in this cohort demands a specific inquiry into the causative factors (Salih et al., 2023).

Several interconnected factors, both universal to medical education and specific to the context, likely contribute to this high prevalence. The pre-clinical and clinical curriculum are intrinsically rigorous, characterized by vast amounts of complex information to be mastered, high-stakes examinations, and a highly competitive environment. This sustained academic pressure can lead to chronic stress, sleep deprivation, and burnout, which are well-established precursors to anxiety and depressive disorders (Malebari et al., 2024). Furthermore, the transition into clinical rotations introduces a unique set of stressors, including direct responsibility for patient care, confronting human suffering and mortality, and navigating complex hierarchical structures within healthcare teams. This transition often triggers “clinical shock,” a phenomenon associated with a significant spike in anxiety and stress levels (Lertanansit et al., 2025).

Beyond these universal challenges, context-specific factors may be at play. The post-pandemic context has generated both residual and emergent stressors, encompassing disruptions to conventional educational modalities, heightened social isolation, and increased anxieties related to health and future professional careers. Studies conducted in the post-2021 period have consistently shown a worsening of mental health metrics among student populations globally, and our findings may reflect this broader trend (Bersia et al., 2024). Additionally, the socio-cultural context of medical education in Saudi Arabia, while rapidly evolving, may still involve significant family and societal expectations regarding academic excellence and career success. This external pressure can internalize as a fear of failure or disappointing one’s family, thereby exacerbating anxiety (Alwhaibi et al., 2023). Institutional culture itself is a critical determinant. A learning environment deficient in supportive infrastructures, characterized by punitive responses to academic underperformance, and lacking initiatives to destigmatize mental health concerns can substantially exacerbate the intrinsic stressors associated with the medical curriculum (Almansour et al., 2024).

The finding that anxiety represented the most severely affected domain, with nearly half of participants classified in the “extremely severe” category, is particularly indicative of the disproportionate burden of this condition. This may be linked to the specific nature of anxiety symptoms measured by the DASS-21, which include autonomic arousal, skeletal muscle effects, and situational anxiety, all of which are readily triggered by the constant anticipation of exams, oral presentations, and clinical evaluations that define medical training (Isha et al., 2023; Piyapinyo et al., 2025). The “extremely severe” classification suggests that for a large portion of students, these symptoms are pervasive and debilitating, likely to impair their academic performance, clinical learning, and overall quality of life.

When compared to a similar study conducted at a public university in Riyadh, which reported a 35% prevalence of severe to extremely severe anxiety, our figure of 44.3% appears elevated (Al-Khlaiwi et al., 2023). This discrepancy could be attributed to sample characteristics, different measurement tools, or unique institutional pressures at a private medical college, such as higher financial costs of education placing additional stress on students and their families. This comparison underscores that the mental health burden is not uniform across all institutions and that localized data, as presented here, is essential for crafting effective, targeted interventions (Jabali, 2025).

In conclusion, the data from this study provide compelling evidence of substantial psychological distress among medical and health sciences students. The prevalence of depression, anxiety, and stress is not only elevated but clinically significant, with anxiety demonstrating epidemic-level severity. This phenomenon reflects a systemic challenge arising from the intersection of academically rigorous curricula, developmental vulnerabilities, and potentially insufficient institutional or cultural support structures. These findings underscore the imperative for college administrations to embed student mental health as a central component of their educational mission, transitioning from reactive crisis management toward the establishment of a sustainable, supportive, and psychologically healthy learning environment.

### Demographic determinants of mental health

#### Gender disparities

A pivotal finding of this study is the significant association between gender and all three domains of psychological distress. Female students consistently reported higher mean scores for depression, anxiety, and stress compared to their male counterparts. This gender disparity is a well-documented phenomenon in the literature on student mental health, both in Saudi Arabia and globally (Gao et al., 2020). The heightened vulnerability among female students may be attributed to a confluence of factors, including greater academic stress perception, higher rates of help-seeking behavior leading to more self-reporting, and sociocultural pressures that are particularly pronounced in the region (Almutairi and Jahan, 2022; Chookerd et al., 2025). Furthermore, female students may face additional stressors related to future career balancing and societal expectations, which can exacerbate psychological distress (Yaghmour et al., 2021).

#### Age and residence as factors

Our analysis also identified age and place of residence as significant demographic determinants. Younger students (18–22 years) reported higher anxiety and stress, which may be linked to the challenges of adjusting to the rigorous demands of medical school (Kebede et al., 2019). Interestingly, the 31–35 age group showed the highest mean stress scores, suggesting that mature students balancing academic commitments with familial or financial responsibilities experience a unique set of stressors (Olson et al., 2025).

Regarding residence, students from urban areas reported significantly higher anxiety than those from rural settings. This finding contrasts with some studies that often associate rural areas with greater distress due to isolation. However, in our context, urban stressors such as a higher cost of living, increased social competition, and sensory overload in city environments might contribute to elevated anxiety levels. The competitive academic atmosphere in an urban college setting could further intensify these feelings (Tuersun et al., 2025).

#### The complex role of academic performance

The association between academic performance, as measured by GPA, and mental health outcomes was complex and multifactorial. While students with “Outstanding” GPAs constituted the largest proportion of those in the “normal” severity category for depression and anxiety, they also represented the largest proportion in the “severe” and “extremely severe” categories. This indicates that high academic achievement is not a protective factor against mental distress and may even be a risk factor for some. This paradox can be explained by the concept of perfectionism, which is prevalent among high-achieving medical students (Atwa et al., 2024). The relentless pursuit of excellence, fear of failure, and intense self-criticism can lead to significant anxiety and depressive symptoms, even in the context of apparent academic success (Shin et al., 2023; Hassan et al., 2025). This finding challenges the assumption that high performers are coping well and highlights the need for support services that address maladaptive perfectionism.

#### Barriers to accessing mental health services

The Barriers to Mental Health Services Scale-Revised (BMHSS-R) demonstrated excellent overall reliability in our sample, indicating its utility for this population. The results showed that barriers were significantly associated with academic variables like major, study year, and GPA, but not with gender or age. This suggests that the perception of obstacles is more closely tied to a student’s academic journey and institutional context than to their demographic profile (Alsalman et al., 2024).

The strong positive correlation between total intrinsic barriers (e.g., stigma, attitudes) and total extrinsic barriers (e.g., cost, transportation) is a critical finding. It implies that students who struggle with internalized stigma are also more likely to perceive external obstacles as insurmountable, and vice-versa (Pepin et al., 2009). This creates a compounded barrier to care. Subscales like “Insurance/payment concerns” and “Beliefs about the inability to find a psychotherapist” had high mean scores, pointing to significant structural and informational hurdles. The persistence of stigma as a barrier, despite its lower Cronbach’s alpha in this study, aligns with regional research emphasizing the role of cultural perceptions of mental illness in hindering service utilization (Alhumaidan et al., 2024). A multi-pronged intervention strategy is therefore essential, one that simultaneously works to destigmatize mental health issues *and* improves the accessibility and affordability of services.

### Implications and future directions

The findings of this study carry significant implications for educational policy and practice. There is an urgent need for medical and health sciences institutions to establish systematic, accessible, and destigmatized mental health support frameworks. Recommended measures include:

- **Targeted outreach initiatives** for high-risk subgroups, such as female students and high-achievers exhibiting perfectionistic tendencies.
- **Institutional campaigns** aimed at reducing stigma surrounding mental health and promoting normalization of help-seeking behaviors.
- **Structural enhancements**, including provision of affordable or free counseling services and clear communication regarding pathways to access mental health resources.

Future research should adopt longitudinal designs to monitor the trajectory of psychological distress over time and employ qualitative methodologies to capture the lived experiences and specific barriers encountered by students. Multi-institutional investigations are also warranted to validate these findings across diverse educational contexts within the Kingdom.

### Limitations

This study has several limitations that necessitate consideration. The cross-sectional design restricts causal interpretation of the associations between sociodemographic factors and mental health consequences among medical students. Moreover, the use of convenience sampling may have introduced selection bias, thereby restricting the generalizability of the findings; probability-based sampling methods would improve representativeness in future research. Although the DASS-21 is a validated and widely used screening questionnaire, it does not provide clinical diagnoses, highlighting the need for complementary diagnostic assessments in subsequent studies. The reliance on self-reported data also introduces the potential for response bias, which could be mitigated through the inclusion of objective or clinician-administered evaluations. Finally, as this study was conducted at a single institution, the results may not fully reflect the experiences of medical students in other contexts. Future multi-institutional and longitudinal studies are recommended to enhance the robustness and generalizability of these findings and to inform targeted mental health interventions for medical students globally.

## Conclusion

This study reveals a significant prevalence of depression, anxiety, and stress among medical and health sciences students, highlighting the urgent need for institutional action. Psychological distress appears to be a widespread issue rooted in the structure and culture of medical education. Factors such as gender, age, residence, and academic performance significantly influence mental health outcomes, underscoring its multifactorial nature. Additionally, intrinsic barriers like stigma and self-reliance, along with extrinsic barriers such as accessibility and cost, hinder students from seeking professional support.

The findings stress that academic achievement should not come at the cost of psychological wellbeing. Institutions must adopt a holistic approach that integrates mental health promotion into medical education through accessible counseling services, destigmatization efforts, mentorship programs, and flexible academic policies prioritizing student welfare.

In conclusion, this research provides essential evidence to inform policy and intervention strategies within Saudi Arabia and similar contexts. Future studies should use longitudinal and multi-institutional approaches to monitor mental health trends and evaluate the effectiveness of targeted initiatives. Addressing both the causes and barriers of psychological distress can help create a supportive environment where future healthcare professionals thrive academically, emotionally, and socially.

## Data Availability

The original contributions presented in the study are included in the article/supplementary material, further inquiries can be directed to the corresponding author.

## Footnotes

1
http://www.raosoft.com/sample-size.html
